# Efficacy of the bleeding risk scoring system for optimal prophylactic anticoagulation therapy of venous thromboembolism in trauma patients: a single-center, retrospective, observational cohort study

**DOI:** 10.1186/s40780-023-00319-5

**Published:** 2023-12-19

**Authors:** Atsushi Tomizawa, Takaaki Maruhashi, Akito Shibuya, Akihiko Akamine, Masayuki Kuroiwa, Yuichi Kataoka, Yasushi Asari, Koichiro Atsuda, Katsuya Otori

**Affiliations:** 1 Department of Pharmacy, Kitasato University Hospital, 1-15-1, Kitasato, Minami-koi, Sagamihara, Kanagawa 252-0373 Japan; 2 Department of Emergency and Critical Care Medicine, Kitasato University School of Medicine, 1-15-1, Kitasato, Minami-Ku, Sagamihara, Kanagawa 252-0373 Japan; 3 Department of Anesthesiology, Kitasato University School of Medicine, 1-15-1, Kitasato, Minami-ku, Sagamihara, Kanagawa 252-0373 Japan; 4 Research and Education Center for Clinical Pharmacy, Division of Clinical Pharmacy, Laboratory of Pharmacy Practice and Science 1, Kitasato University School of Pharmacy, 5-9-1, Shirokane, Minato-ku, Tokyo, 108-8641 Japan

**Keywords:** Venous thromboembolism, Bleeding risk scoring system, Trauma, Prophylactic anticoagulation therapy

## Abstract

**Background:**

We developed a bleeding risk scoring system (BRSS) using prophylactic anticoagulation therapy to comprehensively assess the risk of venous thromboembolism (VTE) in trauma patients. This study evaluated the usefulness of this system in trauma patients, with a focus on minimizing the rate of bleeding events associated with prophylactic anticoagulation therapy.

**Methods:**

We retrospectively evaluated the efficacy of BRSS in trauma patients who received prophylactic anticoagulation therapy for VTE at the Kitasato University Hospital Emergency and Critical Care Center between April 1, 2015, and August 31, 2020. To compare the incidence of bleeding events, patients were divided into two groups: one group using the BRSS (BRSS group) and another group not using the BRSS (non-BRSS group).

**Results:**

A total of 94 patients were enrolled in this study, with 70 and 24 patients assigned to the non-BRSS and BRSS groups, respectively. The major bleeding event rates were not significantly different between the two groups (BRSS group, 4.2%; non-BRSS group, 5.7%; *p* = 1.000). However, minor bleeding events were significantly reduced in the BRSS group (4.2% vs.27.1%; *p* = 0.020). Multivariate logistic regression analysis showed that BRSS was not an independent influencing factor of major bleeding events (odds ratio, 0.660; 95% confidence interval: 0.067-6.47; *p =* 0.721). Multivariate logistic regression analysis showed that BRSS was an independent influencing factor of minor bleeding events (odds ratio, 0.119; 95% confidence interval: 0.015-0.97; *p =* 0.047). The incidence of VTE did not differ significantly between groups (BRSS group, 4.2%; non-BRSS group, 8.6%; *p* = 0.674).

**Conclusions:**

BRSS may be a useful tool for reducing the incidence of minor bleeding events during the initial prophylactic anticoagulation therapy in trauma patients. There are several limitations of this study that need to be addressed in future research.

**Supplementary Information:**

The online version contains supplementary material available at 10.1186/s40780-023-00319-5.

## Background

Venous thromboembolism (VTE) is a common and severe complication of trauma [1, 2]. The risk of VTE is higher in patients with major trauma than in those with minor trauma [3]. Immediately after injury, a hypercoagulable state occurs because of increased generation of thrombin and fibrin [4]. This hypercoagulable state, along with any manifested blood coagulation disorder, increases the risk of VTE. Thrombin levels may increase significantly within 24 h of injury and remain elevated for approximately 5 days [5]. Therefore, prophylaxis for VTE after injury in trauma patients is urgently required.

Prophylaxis for VTE can be mechanical or pharmacological. In Japan, pharmacological prophylaxis involves anticoagulation therapy using unfractionated heparin (UFH), low-molecular-weight heparin (LMWH), Xa factor inhibitors, and vitamin K antagonists. Prophylactic anticoagulation therapy is selected for patients at a high risk of VTE, such as trauma patients. Prophylactic anticoagulation therapy is more effective than mechanical prophylaxis in reducing the risk of deep vein thrombosis (DVT) in trauma patients. However, it also increases the risk of minor bleeding [6]. Inappropriate prophylactic anticoagulation therapy can lead to bleeding. Therefore, it is necessary to appropriately assess the bleeding risk when prophylactic anticoagulation is administered to trauma patients. The International Medical Prevention Registry on Venous Thromboembolism (IMPROVE) bleeding risk tool was used to predict the risk of clinically important bleeding during prophylactic anticoagulation therapy in patients with acute illness [7]. However, this tool has not been validated for use in critically ill patients, including those with trauma, or in the Japanese population. We developed a bleeding risk-scoring system (BRSS) to help prevent bleeding events associated with prophylactic anticoagulation therapy in trauma patients. The BRSS is a clinical decision-making tool that simplifies the selection of prophylactic anticoagulation therapy for VTE. This study aimed to evaluate the usefulness of the BRSS in trauma patients, with a particular focus on minimizing the rates of bleeding events associated with prophylactic anticoagulation therapy.

## Methods

### Ethics approval

This study was approved by the Institutional Review Board for Observation and Epidemiological Study of Kitasato University Medical Ethics Organization (KMEO B19-360). All procedures involving human participants were performed in accordance with the principles of the Declaration of Helsinki. Informed consent was obtained from the Kitasato University School of Medicine website by using an opt-out system. We informed or disclosed to the patients about the conduct of this study and provided them with ample opportunities to opt out. We anonymized all data used in this study.

### Study design and population

**Fig. 1:**
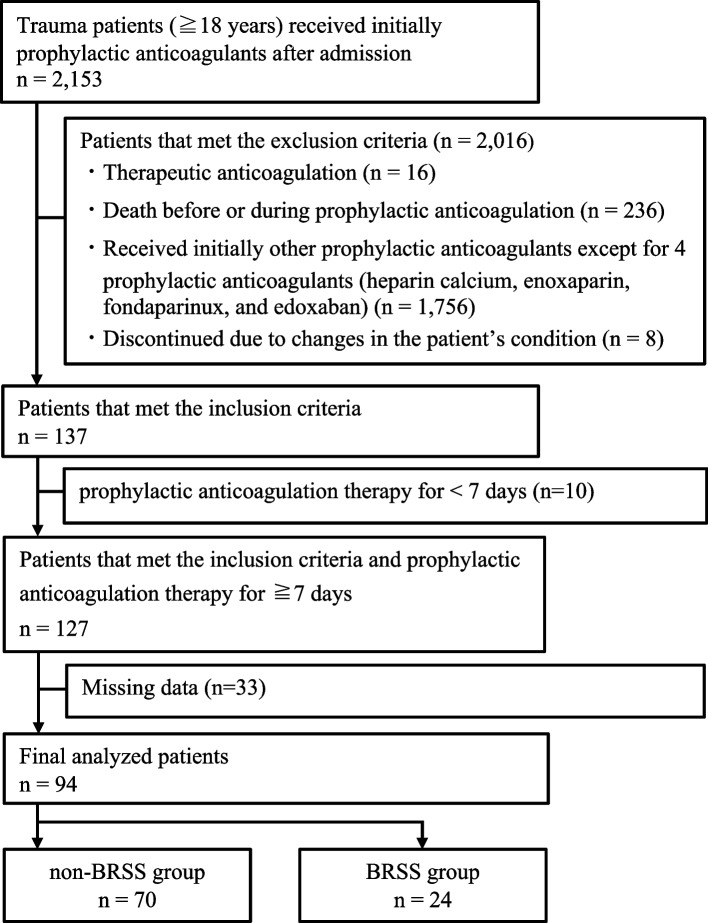
Flowchart of patients’ enrollment. There were 2153 trauma cases from April 1, 2015, to August 31, 2020. A total of 2016 patients met the following exclusion criteria:1) 16 patients received therapeutic anticoagulants for VTE, 2) 236 patients died before or during prophylactic anticoagulation, 3) 1756 patients initially received other prophylactic anticoagulants except for four prophylactic anticoagulants (heparin calcium, enoxaparin, fondaparinux, and edoxaban), and 4) eight patients discontinued the treatment due to changes in their condition after prophylactic anticoagulation therapy; therefore, 137 patients met the inclusion criteria. In addition, from the 137 patients who met the inclusion criteria, we further excluded patients who received prophylactic anticoagulation therapy for < 7 days (*n*  = 10) and those with missing data (*n*  = 33). Finally, 94 patients were enrolled in this study, with 70 and 24 patients assigned to the non-BRSS and BRSS groups, respectively

This was a single-center retrospective observational cohort study. Data were extracted from the hospital information systems. The study population included trauma patients who received prophylactic anticoagulation therapy for VTE at Kitasato University Hospital Emergency and Disaster Medical Center between April 1, 2015, and August 31, 2020. The inclusion criteria were as follows: (1) age ≥ 18 years, (2) received initial prophylactic anticoagulants after admission, and (3) patients who had received initial anticoagulation postoperatively if surgery had been performed. The following patients were excluded: (1) patients receiving therapeutic anticoagulants for VTE (*n* = 16); (2) patients who died before or during prophylactic anticoagulation therapy (*n* = 236); (3) patients who initially received other anticoagulants, except for four prophylactic anticoagulants (heparin calcium, enoxaparin, fondaparinux, and edoxaban) (*n* = 1756); and (4) patients who discontinued prophylactic anticoagulation therapy due to changes in the patient’s condition after commencing prophylactic anticoagulation therapy (*n* = 8). In addition, among the 137 patients who met the inclusion criteria, we further excluded patients who received prophylactic anticoagulation therapy for < 7 days (*n* = 10) and those with missing data (*n* = 33). Finally, 94 patients were enrolled in this study, with 70 and 24 patients assigned to the non-BRSS and BRSS groups, respectively (Fig. 1). The BRSS group was defined as the BRSS group at the time of initial prophylactic anticoagulation therapy because the BRSS was implemented in a template on the electronic medical record.

### VTE risk assessment

In our hospital, we used a computerized clinical decision support system (CCDSS) to assess the risk factors for VTE, including background factors, surgical type, and patient history [8]. CCDSS provides appropriate prophylaxis for VTE according to the VTE risk level. We assessed VTE prophylaxis at the time of admission, time of change in prophylaxis, before the start and end of prophylactic anticoagulation therapy, and further selected prophylaxis according to the VTE risk level. Mechanical prophylaxis is recommended when the risk level is low or moderate. When the VTE risk level is high, prophylactic anticoagulation therapy is recommended in addition to mechanical prophylaxis. When the VTE risk level is highest, both mechanical prophylaxis and prophylactic anticoagulation therapy are recommended. Prophylactic anticoagulation therapy was administered to all the patients if there were no contraindications. Mechanical prophylaxis, such as intermittent pneumatic compression (IPC) or compression stockings, has also been used. DVT was defined as a new thrombus within the venous system after admission, and was recorded after confirmation by vascular ultrasonography. Vascular ultrasonography was performed by a clinical laboratory technician and the results were evaluated by emergency department doctors.

### Bleeding risk assessment

**Fig. 2:**
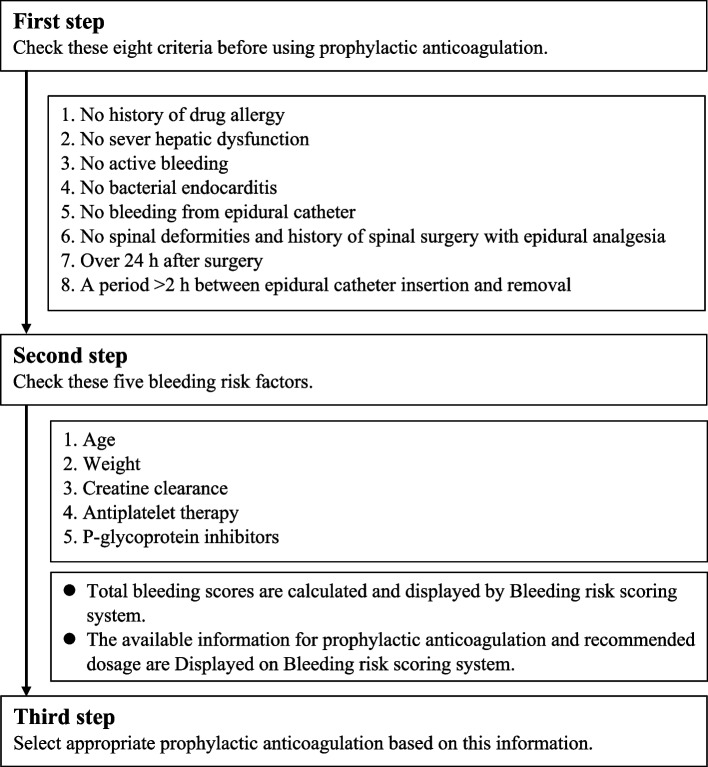
Bleeding risk scoring system. The bleeding risk scoring system is an auto-calculation system for the bleeding risk scores. In the first step, we checked eight criteria: complications and precautions for prophylactic anticoagulation therapy. In the second step, we assessed the five bleeding risk factors. The bleeding risk scoring system calculates the bleeding risk score and displays the available information on prophylactic anticoagulation and the recommended dosage of prophylactic anticoagulation therapy. Finally, we selected appropriate prophylactic anticoagulation based on this information

We created bleeding risk criteria associated with bleeding risk factors in other studies [7, 9, 10]. The bleeding risk score was determined based on bleeding risk factors associated with prophylactic anticoagulation therapy. We determined that the score would be higher if there were contraindications or if there was no experience with contraindicated medications, as the importance of avoiding these drugs was emphasized. We adjusted the distribution of BRSS to consider more than one dominant factor (Table 1). The BRSS was administered by emergency department doctors at the time of the initial prophylactic anticoagulation therapy after admission (Fig. 2). First, we evaluated eight contraindications and precautions to be considered prior to initial prophylactic anticoagulation therapy. Second, we examined five bleeding risk factors: age [7, 9, 10], body weight [11], creatinine clearance [10, 12], antiplatelet therapy [10], and P-glycoprotein inhibitors [11, 13]. The BRSS automatically calculates the bleeding risk score and displays the available information for prophylactic anticoagulation and the recommended dosage of prophylactic anticoagulation therapy. Finally, appropriate prophylactic anticoagulation was selected based on this information (Table 2). The following prophylactic anticoagulants were used: (1) UFH (Heparin calcium; Sawai); 5000 U twice daily subcutaneously, (2) enoxaparin (Clexane; Sanofi); 20 mg once or twice daily subcutaneously, (3) fondaparinux (Arixtra; Novartis); 1.5 mg or 2.5 mg once daily subcutaneously, (4) edoxaban (Lixiana; Daiichi-Sankyo); 15 mg or 30 mg once daily orally.

**Table 1:** Bleeding risk scores for prophylactic anticoagulation

Bleeding risk factor	Heparin calcium (s.c.)	Enoxaparin (s.c.)	Fondaparinux (s.c.)	Edoxaban (p.o.)
Age (years)				
≥ 75	1	1	1	1
< 75	0	0	0	0
Weight (kg)				
≥ 50	0	0	0	0
≥ 40, < 50	1	1	1	1
< 40	3	3	3	3
CLcr (mL/min)				
≥ 50	0	0	0	0
≥ 30, < 50	0	1	1	1
≥ 20, < 30	0	5	1	5
< 20	5	5	6	5
Antiplatelet therapy				
None	0	0	0	0
SAPT	Physician consultation	Physician consultation	Physician consultation	Physician consultation
DAPT	Physician consultation	Physician consultation	Physician consultation	Physician consultation
P-gp inhibitors				
None	0	0	0	0
1	0	0	0	1
≥ 2	0	0	0	2

Abbreviations: *s.c.* subcutaneous injection, *p.o* per OS, *CLcr* creatinine clearance, *SAPT* single antiplatelet therapy, *DAPT* dual antiplatelet therapy, *P-gp inhibitors*, p-glycoprotein inhibitors (quinidine, verapamil, amiodarone, cyclosporine, erythromycin, itraconazole)

**Table 2:** Bleeding risk scores and recommendation for prophylactic anticoagulation

	Recommendation for prophylactic anticoagulation			
Bleeding risk score	Heparin calcium (s.c.)	Enoxaparin (s.c.)	Fondaparinux (s.c.)	Edoxaban (p.o.)
0	5000 U twice a day	2000 IU twice a day	2.5 mg once a day	30 mg once a day
1–4	5000 U twice a day	2000 IU once a day	1.5 mg once a day	15 mg once a day
5	5000 U twice a day	Not recommended	1.5 mg once a day	Not recommended
6–11	Not recommended or 5000 U twice a day (only if needed)	Not recommended	Not recommended	Not recommended

Abbreviations: *s.c.* subcutaneous injection, *p.o.* per OS

### Data collection

Data on age, sex, body weight, acute physiology and chronic health evaluation II (APACHE-II) score, sequential organ failure assessment (SOFA) score, injury severity score (ISS), major injury site, and complications were collected at the time of admission to the emergency and disaster medical center. Blood transfusions were collected during prophylactic anticoagulation therapy, if blood transfusions were performed. IPC or compression stockings for mechanical prophylaxis were collected between admission and discharge. Data on hemoglobin (Hb), platelet, creatinine clearance (CLcr), total bilirubin, aspartate aminotransferase, and alanine aminotransferase levels were collected before the commencement of prophylactic anticoagulation therapy.

### Outcome

The primary outcome was the rate of major bleeding events. Major bleeding events were defined as a decline in Hb to > 2 g/dL [14], bleeding from the digestive organs, and gastrointestinal bleeding due to the high frequency of gastrointestinal bleeding with anticoagulants such as direct oral anticoagulants [15]. The secondary outcomes were the rate of minor bleeding events, occurrence of VTE, rate of inappropriate prophylactic anticoagulation therapy, length of stay in the emergency center, length of hospital stay, and duration of prophylactic anticoagulation therapy. Minor bleeding events were classified as instances of hematuria or a shift from negative latent urinary blood before prophylactic anticoagulation to positive latent urinary blood during or after prophylactic anticoagulation. Minor bleeding events were defined based on the Bleeding Academic Research Consortium Definition for Bleeding (Type2 events) using A Consensus Report From the Bleeding Academic Research Consortium [16] and a previous study [17]. Inappropriate prophylactic anticoagulation therapy was defined as the use of contraindicated therapy, simultaneous administration of prophylactic anticoagulants with contraindicated medicines, off-label use, or overdosage or dosage of anticoagulants. The follow-up period for bleeding events was from the start of anticoagulant administration to the day after the end of administration. The follow-up period for VTE was from the time of admission to the hospital until the end of the VTE prophylactic anticoagulation therapy.

### Main analysis

This study aimed to identify the effect of BRSS on major and minor bleeding events in trauma patients. Therefore, the effect of BRSS including APACHE-II score and ISS as explanatory variables on major or minor bleeding events was analyzed using multivariate logistic regression models in 94 patients finally enrolled in this study.

### Sensitivity analysis

We also performed the following analysis to consider the differences in the analysis population or imbalances in patient characteristics.

1. The effect of BRSS on major or minor bleeding events was analyzed using univariate logistic regression models in 137 patients that met the inclusion criteria with multiple imputations for missing values.
2. The effect of BRSS on major or minor bleeding events was analyzed using univariate logistic regression models in 127 patients that met the inclusion criteria and prophylactic anticoagulation therapy for more than 7 days with multiple imputations for missing values.
3. The effect of BRSS on major or minor bleeding events was analyzed using univariate logistic regression models in 122 patients that met the inclusion criteria and were excluded from the missing value of bleeding events.
4. The effect of BRSS, including APACHE-II score and ISS as explanatory variables, on major or minor bleeding events was analyzed using multivariate logistic regression models in 137 patients that met the inclusion criteria with multiple imputations for missing values.

### Statistical analysis

Fisher’s exact test was used to compare categorical variables between the BRSS and non-BRSS groups, while the Mann-Whitney U test was used to analyze continuous variables. Categorical data were presented as absolute values and percentages of the population, and quantitative data were presented as medians and interquartile ranges. Categorical and continuous variables were compared between the BRSS and non-BRSS groups, excluding patients with missing data from the primary analysis. However, considering the missing values in the sensitivity analysis, multiple imputations were performed using chained equations to create 100 sets of multiple imputation data in R version 4.2.2(R Development Core Team) after a Multivariate Imputation via Chained Equations (MICE) package was installed and loaded into the R library. All statistical analyses were conducted using EZR version 1.61 software (Saitama Medical Center, Jichi Medical University, Saitama, Japan) and a graphical user interface for R version 4.2.2, a modified R Commander version designed to add statistical functions frequently used in biostatistics [18]. All tests were two tailed. Statistical significance was set at *p* < 0.05. *P*-values for non-primary outcomes were nominal, to account for multiplicity.

## Results

### Patient characteristics

Table 3 shows the patient characteristics of the BRSS and non-BRSS groups. The median age of the patients was 40 years. There was no significant difference in age between the BRSS and non-BRSS groups. The APACHE-II and SOFA scores and ISS were also not significantly different between the BRSS and non-BRSS groups. The limbs and pelvis were the most common sites of injury in both the groups, accounting for > 50% of all injuries. IPC (45.7%) or compression stockings (54.3%) were used as mechanical prophylaxis in all patients. The rates of IPC and compression stockings in the BRSS and non-BRSS groups are shown in Table 3. The mechanical prophylaxis rate did not differ significantly between the BRSS and non-BRSS groups. Although the rate of prophylactic anticoagulation initiation within 48 h after admission was not significantly different between the BRSS and non-BRSS groups (20.8% vs. 17.1%; *p* = 0.760), the time of initiation of prophylactic anticoagulation after admission was earlier in the BRSS group than in the non-BRSS group (4.5 days vs. 9.0 days; *p* = 0.024).

**Table 3:** Patients’ characteristics

	All patients	BRSS group	non-BRSS group	*p*-value
	*n* = 94	*n* = 24	*n* = 70	
Age, years	43	40	44	0.202
median (IQR)	(28.0–61.0)	(26.0–54.0)	(33.0–66.0)	
Sex, *n* (%)				0.322
Male	61 (64.9)	18 (75.0)	43 (61.4)	
Female	33 (35.1)	6 (25.0)	27 (38.6)	
Weight, kg	69	70.8	67.2	0.276
median (IQR)	(55.3–77.6)	(60.9–79.1)	(54.6–77.3)	
APACHE-II score	15	14	15	0.546
median (IQR)	(11.0–21.0)	(11.0–20.0)	(12.0–21.0)	
SOFA score	3	3	3	0.672
median (IQR)	(1.0–4.0)	(2.0–3.0)	(1.0–4.0)	
ISS	22	18	24.5	0.062
median (IQR)	(13.0–34.0)	(9.0–34.0)	(14.5–34.8)	
Major injury site, *n* (%)				0.485
Head and neck	21 (22.3)	7 (29.2)	14 (20.0)	
Chest	21 (22.3)	6 (25.0)	15 (21.4)	
Abdomen	2 (2.1)	1 (4.2)	1 (1.4)	
Limb and pelvis	50 (53.2)	10 (41.7)	40 (57.1)	
Complicated injuries, *n* (%)	47 (50.0)	10 (41.7)	37 (52.9)	0.478
Blood transfusion, *n* (%)	2 (2.1)	0 (0.0)	2 (2.9)	1.000
Mechanical prophylaxis, *n* (%)				0.354
IPC	43 (45.7)	13 (54.2)	30 (42.9)	
Compression stockings	51 (54.3)	11 (45.8)	40 (57.1)	
Prophylactic anticoagulation, *n* (%)				0.249
Fondaparinux	55 (58.5)	12 (50.0)	43 (61.4)	
Heparin calcium	30 (31.9)	11 (45.8)	19 (27.1)	
Edoxaban	9 (9.6)	1 (4.2)	8 (11.4)	
^a^Started within 48 h, *n* (%)	17 (18.1)	5 (20.8)	12 (17.1)	0.760
Start day of initial prophylactic anticoagulation from admission	8	4.5	9	0.024
median (IQR)	(3.3–14.0)	(3.0–9.0)	(4.3–15.8)	
Laboratory data on pre-prophylactic anticoagulation				
median (IQR)				
Hb (g/dL)	9.5	10.2	9.4	0.052
	(8.6–11.4)	(8.8–12.9)	(8.6–10.8)	
Plt (10^4 /μL)	24.2	22.1	25.9	0.273
	(17.8–34.6)	(18.3–26.6)	(17.6–36.4)	
T-Bil (mg/dL)	0.8	0.8	0.8	0.771
	(0.6–1.2)	(0.6–1.3)	(0.6–1.2)	
AST (IU/L)	36	33	37	0.585
	(26.3–68.8)	(26.8–60.0)	(25.5–72.5)	
ALT (IU/L)	34.5	30.5	35	0.493
	(22.0–74.8)	(22.5–57.0)	(22.0–84.3)	
CLcr (mL/min)	143.4	145.9	139.5	0.677
	(117.0–164.5)	(123.4–172.5)	(108.9–163.4)	

*APACHE* acute physiology and chronic health evaluation, *SOFA* sequential organ failure assessment, *IPC* intermittent pneumatic compression, *ISS* injury severity score, *IQR* interquartile range, *Hb* hemoglobin, *Plt* platelet, *T-Bil* total bilirubin, *AST* aspartate aminotransferase, *ALT* alanine aminotransferase, *CLcr* creatinine clearance (simulated Cockcroft-Gault formula)

^a^prophylactic anticoagulation started within 48 h after admission

### Outcomes

The major bleeding event rates were not significantly different between the BRSS and non-BRSS groups (4.2% vs. 5.7%, *p* = 1.000). Minor bleeding event rates were significantly lower in the BRSS group than in the non-BRSS group (4.2% vs. 27.1%; *p* = 0.020). The incidence of VTE did not differ significantly between groups (4.2% vs. 8.6%; *p* = 0.674). The duration of prophylactic anticoagulation therapy was significantly shorter in the BRSS group than in the non-BRSS group (11 days vs. 14 days, *p* = 0.015). The rates of inappropriate prophylactic anticoagulation, length of stay in the emergency center, and length of hospital stay did not differ significantly between the two groups (Table 4).

**Table 4:** Outcomes in the BRSS and non-BRSS groups

	BRSS group	non-BRSS group	*p*-value
	*n* = 24	*n* = 70	
Major bleeding events, *n* (%)	1 (4.2)	4 (5.7)	1.000
Minor bleeding events, *n* (%)	1 (4.2)	19 (27.1)	0.020
VTE, *n* (%)	1 (4.2)	6 (8.6)	0.674
Inappropriate use, *n* (%)	2 (8.3)	12 (17.1)	0.507
Contraindication, *n* (%)	0 (0)	0 (0)	NA
Contraindicated medicines, *n* (%)	0 (0)	0 (0)	NA
Off-label use, *n* (%)	2 (8.3)	9 (12.9)	0.723
Under dosing, *n* (%)	0 (0)	2 (2.9)	1.000
Over dosing, *n* (%)	0 (0)	1 (1.4)	1.000
Length of stay in emergency center, median (IQR)	17.5 (13.5–39.0)	19.50 (14.3–32.8)	0.696
Hospital length of stay, median (IQR)	43.5 (28.5–72.8)	48.5 (36.0–59.8)	0.955
The duration of prophylactic anticoagulation, median (IQR)	11.5 (7.0–14.0)	14.0 (11.3–14.0)	0.004

*IQR* interquartile range, *VTE* venous thromboembolism

### Main analysis result

Multivariate logistic regression analysis showed that BRSS was not an independent influencing factor of major bleeding events (odds ratio, 0.660; 95% confidence interval: 0.067-6.47; *p =* 0.721). Multivariate logistic regression analysis showed that BRSS was an independent influencing factor for minor bleeding events (odds ratio, 0.119; 95% confidence interval: 0.015-0.97; *p* = 0.047) (Table 5).

**Table 5:** The effect of BRSS including APACHE-II score and ISS as explanatory variables on major or minor bleeding events using multivariate logistic regression models in 94 patients finally enrolled in this study (*n* = 94)

	Risk factor	Odds ratio	95%CI	*P value*
Major bleeding	BRSS	0.660	0.067 - 6.470	0.721
	APACHE-II score	0.973	0.846 - 1.120	0.696
	ISS	0.989	0.918 - 1.070	0.774
Minor bleeding	BRSS	0.119	0.015 - 0.971	0.047
	APACHE-II score	1.080	1.000 - 1.160	0.041
	ISS	1.010	0.969 - 1.050	0.647

Abbreviations: *CI* confidence interval, *BRSS* Bleeding risk scoring system, *APACHE* Acute physiology and chronic health evaluation, *ISS* Injury severity score

### Sensitivity analysis result

1. Univariate logistic regression analysis of 137 patients that met the inclusion criteria with multiple imputations for missing values showed that BRSS was not an independent influencing factor for major bleeding events (odds ratio: 0.504; 95% confidence interval: 0.054-4.671; *p =* 0.544); however, BRSS was an independent influencing factor for minor bleeding events (odds ratio: 0.189; 95% confidence interval: 0.041-0.878; *p =* 0.034) (Supplementary Table 1).
2. Univariate logistic regression analysis of 127 patients met the inclusion criteria, and prophylactic anticoagulation therapy for more than 7 days with multiple imputations for missing values showed similar results for major bleeding events (odds ratio: 0.492; 95% confidence interval: 0.052-4.637; *p =* 0.533) and minor bleeding events (odds ratio: 0.185; 95% confidence interval: 0.039-0.858; *p* = 0.031) (Supplementary Table 2).
3. Univariate logistic regression analysis of 122 patients met the inclusion criteria and were excluded from missing bleeding event values showed similar results for major bleeding events (odds ratio: 0.548; 95% confidence interval: 0.060-4.992; *p =* 0.591) and minor bleeding events (odds ratio: 0.206; 95% confidence interval: 0.045-0.947; *p =* 0.042) (Supplementary Table 3).
4. Multivariate logistic regression analysis of 137 patients that met the inclusion criteria with multiple imputation for missing values showed similar results for major bleeding events (odds ratio: 0.503; 95% confidence interval: 0.053-4.816; *p =* 0.548) and minor bleeding events (odds ratio: 0.185; 95% confidence interval: 0.038-0.910; *p =* 0.038) (Supplementary Table 4).

## Discussion

The BRSS did not significantly reduce the incidence of major bleeding events. However, the incidence of minor bleeding events was significantly lower in the BRSS group than that in the non-BRSS group. BRSS was an independent factor influencing minor bleeding events. These results were also similar in the sensitivity analysis to consider the differences in the analysis population or imbalances in patient characteristics. This study demonstrated the utility of BRSS, which may lead to the safe use of prophylactic anticoagulation therapy in trauma patients struggling with bleeding. This was presumably because the BRSS was created with a point gradient for bleeding risk and the highest priority was given to avoid bleeding complications. A comprehensive assessment of bleeding risk using the BRSS may add several values to the discretion of emergency department doctors involved in trauma care who have difficulty controlling bleeding events, because abnormalities in the coagulation and fibrinolytic systems make it difficult to control bleeding in trauma patients. The BRSS allows emergency department doctors to quantitatively and qualitatively assess several bleeding risks and standardize applicable criteria, including medications, dosage, treatment timing, and duration of administration. Therefore, the BRSS may be a useful tool for guiding the introduction of prophylactic anticoagulation therapy in trauma patients.

Minor bleeding events have been previously reported in 4.1% of patients who received fondaparinux as a prophylactic anticoagulation therapy after hip fracture surgery [19]. Non-major bleeding events have been previously reported in 13.2% of critically ill patients who received UFH as prophylactic anticoagulation therapy [20]. A recent real-world study reported minor bleeding events in 9.3% of patients who received fondaparinux or enoxaparin as anticoagulation therapy for symptomatic VTE [21]. The rate of minor bleeding events observed in this study was higher than that observed in the previous study, which may be attributed to patients presenting with trauma and a difference in the definition of bleeding. There is a paucity of data on bleeding events associated with prophylactic anticoagulation therapy in trauma patients; therefore, a direct comparison of our data with other data is not possible, as the patient populations and their susceptibility to bleeding may differ.

Prophylactic anticoagulation therapy for VTE is limited to patients with severe traumas. The NICE guidelines [22] state that prophylactic anticoagulation therapy for VTE in patients with severe trauma should be administered immediately after risk assessment when the risk of VTE is higher than that of bleeding. Prophylactic anticoagulation therapy within 24 h is recommended for trauma patients who have a moderate or high VTE risk without active bleeding [23]. Prophylactic anticoagulation therapy within 48 h of administration is possible in patients with severe trauma or blunt head trauma with combined acute subdural and subarachnoid hemorrhage [24–27]. However, the initial timing of prophylactic anticoagulation therapy after administration differs among hospitals [28]. In this study, the rate of prophylactic anticoagulation therapy within 48 h after admission was 18.1% because patients who received early prophylactic anticoagulants, such as heparin sodium, were excluded. A recent guideline [29] showed that the initial timing of prophylactic anticoagulation therapy after admission in traumatic brain injury should be decided individually based on multiple factors, including injury severity. Patients with traumatic brain injury accounted for 20% of the total patients; however, the damage to other areas was relatively severe (ISS, median:22). Therefore, the bleeding was difficult to control, and the initial timing of prophylactic anticoagulation therapy after admission was delayed. Prophylactic anticoagulation therapy with LMWH may be superior in patients with VTE, traumatic brain injury, or blunt solid organ injury. However, there are no high-quality data regarding the superiority of UFH or LMWH with regard to traumatic brain injury or blunt solid organ injury specifically [29]. Although UFH or LMWH as prophylactic anticoagulation therapy for VTE was used in critically ill patients, there was no significant difference in the rates of major bleeding events between UFH and LMWH [20]. However, there is no evidence of a difference between subcutaneous and intravenous UFH for preventing VTE recurrence, VTE-related or all-cause mortality, and major bleeding [30]. There is also no evidence of a difference between subcutaneous UFH and LMWH [30]. The off-label use of fondaparinux for heparin-induced thrombocytopenia has been reported to be effective and safe in the United States. There were fewer bleeding events in fondaparinux than in other alternatives [31]. Therefore, fondaparinux may be the first-line treatment for patients with previously reported heparin-induced thrombocytopenia. There is no evidence of the best choice for initial prophylactic anticoagulation therapy in trauma patients [29]. In our study, approximately half of all patients experienced trauma to the lower limbs and pelvis and were administered fondaparinux or heparin calcium. Enoxaparin was not administered to avoid the use of off-label medications as a prophylactic anticoagulation therapy for VTE. The difference in the duration of prophylactic anticoagulation therapy may have been influenced by differences in the prophylactic anticoagulants selected. Heparin calcium was used more frequently in the BRSS group than in the non-BRSS group, and the recommended duration of heparin calcium use was shorter than that of the other anticoagulants. There were no criteria for the discontinuation of prophylactic anticoagulation therapy because it was left to the physician’s judgment. It is possible that the more accurately the risk of bleeding was assessed by BRSS, the more accurately the risk of VTE was also assessed, leading to earlier and shorter durations of prophylactic anticoagulation therapy.

This study has several limitations. First, this was a single-center retrospective observational study that used a small patient cohort. Second, many patients who did not meet the inclusion criteria and were excluded from the study raised concerns about the potential for selection bias. Patients who died before or during prophylactic anticoagulation therapy were excluded and only those who survived were analyzed. Since we evaluated bleeding events, patients at a high risk for bleeding who would have been considered for outcomes if they had survived should have also been evaluated. Patients who received prophylactic anticoagulation therapy for < 7 days were also excluded because prophylactic anticoagulation therapy for VTE prophylaxis is recommended for 7-14 days. However, the short-term bleeding events may have been overlooked. For missing data, we completed the missing values in multiple imputations and performed a sensitivity analysis to ensure the robustness of the results. It is possible that the patients’ backgrounds may differ from those of the excluded patients. These points should be clarified in future research. Third, the rate of prophylactic anticoagulation therapy within 48 h of administration was 18.1% in this study because patients who received only four prophylactic anticoagulants (heparin calcium, enoxaparin, fondaparinux, and edoxaban) were included. Intravenous heparin sodium is often used as the initial anticoagulation therapy in trauma patients at the Kitasato University Hospital Emergency and Critical Care Center. The rate of prophylactic anticoagulation therapy within 48 h of administration might have improved slightly if patients with heparin sodium were included. Fourth, enoxaparin was not used as a prophylactic anticoagulant for VTE because it has only been approved for use in abdominal surgery, artificial joint replacement, and hip fracture surgery in Japan. More than half of the patients in this study experienced trauma to their lower limbs and pelvis. Future research including LMWH is necessary to determine the optimal prophylactic anticoagulation therapy for trauma patients. Fifth, the bleeding risk factors in BRSS were age, body weight, CLcr, antiplatelet therapy, and p-glycoprotein inhibitors. These were selected based on the pharmacokinetics and pharmacology of the prophylactic anticoagulation therapy. Other bleeding risks were not assessed; hence, each patient should be carefully assessed for bleeding and thrombus risks. Heparin sodium was also excluded from the BRSS. We plan to further improve BRSS based on these recommendations to enhance its usefulness. Finally, this study did not use the risk assessment profile for thromboembolism (RAPT) score [32]. The RAPT score correctly identifies trauma patients at increased risk of developing DVT [33]. A study found that UFH with intermittent pneumatic compression resulted in a lower incidence of VTE than intermittent pneumatic compression alone in trauma patients and an RAPT score of 5 [34]. Further studies using the RAPT score for VTE and the BRSS for bleeding events are needed in trauma patients.

This study was quite limited in terms of generalizability; BRSS may be a useful tool to reduce the incidence of minor bleeding events for initial prophylactic anticoagulation therapy in patients whose intravenous route is not available and in patients with heparin-induced thrombocytopenia and heparin allergy. BRSS should be verified in future studies.

## Conclusions

BRSS may be a useful tool to reduce the incidence of minor bleeding events during the initial prophylactic anticoagulation therapy in trauma patients. There are several limitations of this study that need to be addressed in future research.

### Supplementary Information

**Additional file 1: Supplementary Table 1.** The effect of BRSS on major or minor bleeding events using univariate logistic regression models in patients met the inclusion criteria with multiple imputations for missing values (*n*=137).

**Additional file 2: Supplementary Table 2.** The effect of BRSS on major or minor bleeding events using univariate logistic regression models in patients met the inclusion criteria and prophylactic anticoagulation therapy for more than 7 days with multiple imputations for missing values (*n*=127).

**Additional file 3: Supplementary Table 3.** The effect of BRSS on major or minor bleeding events using univariate logistic regression models in patients met the inclusion criteria and excluded from missing value of bleeding events (*n*=122).

**Additional file 4: Supplementary Table 4.** The effect of BRSS including APACHE-II score and ISS as explanatory variables on major or minor bleeding events using multivariate logistic regression models in patients met the inclusion criteria with multiple imputations for missing values (*n*=137).

## Data Availability

The datasets generated and analyzed during the current study are available from the corresponding author upon reasonable request.

## Abbreviations

APACHE: Acute physiology and chronic health evaluation
BRSS: Bleeding risk scoring system
CCDSS: Computerized clinical decision support system
CLcr: Creatinine clearance
DVT: Deep vein thrombosis
Hb: Hemoglobin
IPC: Intermittent pneumatic compression
ISS: Injury severity score
LMWH: Low molecular weight heparin
RAPT: Risk assessment profile for thromboembolism
SOFA: Sequential organ failure assessment
UFH: Unfractionated heparin
VTE: Venous thromboembolism
