# Supplementary material for: Efficacy of the bleeding risk scoring system for optimal prophylactic anticoagulation therapy of venous thromboembolism in trauma patients: a single-center, retrospective, observational cohort study
Source: J Pharm Health Care Sci. 2023 Dec 19;9:48. doi: 10.1186/s40780-023-00319-5 (PMC10729339; doi:10.1186/s40780-023-00319-5)
Supplement: Supplementary file 2 — Additional file 2: Supplementary Table 2. The effect of BRSS on major or minor bleeding events using univariate logistic regression models in patients met the inclusion criteria and prophylactic anticoagulation therapy for more than 7 days with multiple imputations for missing values (n=127). [file 40780_2023_319_MOESM2_ESM.docx]

| Supplementary Table 2 The effect of BRSS on major or minor bleeding events using univariate logistic regression models in patients met the inclusion criteria and prophylactic anticoagulation therapy for more than 7 days with multiple imputations for missing values (n=127) | | | | |
| --- | --- | --- | --- | --- |
| **Major bleeding** | Risk factor | Odds ratio | 95%CI | *P value* |
|  | BRSS | 0.492 | 0.052 - 4.637 | 0.533 |
| **Minor bleeding** | Risk factor | Odds ratio | 95%CI | *P value* |
|  | BRSS | 0.185 | 0.039 - 0.858 | 0.031 |
| Abbreviations: CI, confidence interval, BRSS, Bleeding risk scoring system | | | | |
