# Supplementary material for: Efficacy of the bleeding risk scoring system for optimal prophylactic anticoagulation therapy of venous thromboembolism in trauma patients: a single-center, retrospective, observational cohort study
Source: J Pharm Health Care Sci. 2023 Dec 19;9:48. doi: 10.1186/s40780-023-00319-5 (PMC10729339; doi:10.1186/s40780-023-00319-5)
Supplement: Supplementary file 4 — Additional file 4: Supplementary Table 4. The effect of BRSS including APACHE-II score and ISS as explanatory variables on major or minor bleeding events using multivariate logistic regression models in patients met the inclusion criteria with multiple imputations for missing values (n=137). [file 40780_2023_319_MOESM4_ESM.docx]

| Supplementary Table 4 The effect of BRSS including APACHE2 score and ISS as explanatory variables on major or minor bleeding events using multivariate logistic regression models in patients met the inclusion criteria with multiple imputations for missing values (n=137) | | | | |
| --- | --- | --- | --- | --- |
| **Major bleeding** | Risk factor | Odds ratio | 95%CI | *P value* |
|  | BRSS | 0.503 | 0.053 - 4.816 | 0.548 |
|  | APACHE-Ⅱ score | 0.986 | 0.864 - 1.125 | 0.828 |
|  | ISS | 0.999 | 0.929 - 1.073 | 0.974 |
| **Minor bleeding** | Risk factor | Odds ratio | 95%CI | *P value* |
|  | BRSS | 0.185 | 0.038 - 0.910 | 0.038 |
|  | APACHE-Ⅱ score | 1.097 | 1.024 - 1.174 | 0.008 |
|  | ISS | 1.008 | 0.968 - 1.051 | 0.686 |
| Abbreviations: CI, confidence interval, BRSS, Bleeding risk scoring system APACHE, Acute physiologic assessment and chronic health evaluation ISS, Injury severity score | | | | |
